# Effects of Periodontal Treatment in Patients with Periodontitis and Kidney Failure: A Pilot Study

**DOI:** 10.3390/ijerph19031533

**Published:** 2022-01-29

**Authors:** Wen-Chen Chung, Chih-Chin Kao, Chiung-Fang Huang, Chang-Yu Lee, Hsein-Kun Lu, Mai-Szu Wu

**Affiliations:** 1 School of Dentistry, College of Oral Medicine, Taipei Medical University, Taipei 110, Taiwan; chungology@gmail.com (W.-C.C.); jackson@tmu.edu.tw (H.-K.L.); 2 Division of Periodontics, Department of Dentistry, Taipei Medical University Hospital, Taipei 110, Taiwan; m8504005@tmu.edu.tw; 3 Division of Periodontics, Department of Dentistry, Shuang Ho Hospital, Taipei Medical University, New Taipei City 235, Taiwan; 4 Division of Nephrology, Department of Internal Medicine, Taipei Medical University Hospital, Taipei 110, Taiwan; salmonkao@gmail.com; 5 Division of Nephrology, Department of Internal Medicine, School of Medicine, College of Medicine, Taipei Medical University, Taipei 110, Taiwan; 6 Taipei Medical University-Research Center of Urology and Kidney (TMU-RCUK), Taipei Medical University, Taipei 110, Taiwan; 7 Division of Family and Operative Dentistry, Department of Dentistry, Taipei Medical University Hospital, Taipei 110, Taiwan; 8 School of Dental Technology, College of Oral Medicine, Taipei Medical University, Taipei 110, Taiwan; 9 Division of Nephrology, Department of Internal Medicine, Shuang Ho Hospital, Taipei Medical University, New Taipei City 235, Taiwan

**Keywords:** non-surgical periodontal treatment, renal dialysis, kidney failure

## Abstract

Periodontitis and chronic kidney disease are both chronic inflammatory diseases and share some common risk factors. This 3-month pilot study aimed to clarify whether non-surgical periodontal therapy is beneficial in clinical, biochemical, and microbiological conditions in patients with periodontitis and kidney failure. Kidney failure patients with moderate to severe periodontitis were recruited from two hospitals. Treatment group received non-surgical periodontal therapy, and control group received oral hygiene instruction only. Outcome assessments were conducted 1 and 3 months after treatment. Non-parametric tests were used to analyze the patient-level data. Periodontal site-level assessments were analyzed by Student *t*-test and paired *t*-test. Statistical significance was set at *p*-value < 0.05. A total of 11 subjects completed the study. There was no significant difference between groups in all-cause mortality, cardiovascular events, infection events, systemic parameters, and serum biomarkers. Comparing to control group, clinical periodontal parameters, gingival crevicular fluid interleukin-1β (IL-1β) level and periodontal pathogens showed significant improvement in the treatment group. Non-surgical periodontal treatment did not change systemic outcomes in kidney failure patients, but changed the local micro-environment.

## 1. Introduction

Periodontitis is a chronic inflammatory disease which causes loss of periodontal tissues by bacterial infection [1]. Periodontal pathogens, such as *Porphyromonas gingivalis* (*P. gingivalis*), *Treponema denticola* (*T. denticola*), and *Tannerella forsythia* (*T. forsythia*), accumulating on tooth surfaces may not only induce local but systemic inflammatory reaction [2,3]. The eliciting of systemic pro-inflammatory cytokine production originating from the periodontal region may also aggravate the severity of systemic diseases including cardiovascular disease (CVD), diabetes, chronic kidney disease (CKD), rheumatoid arthritis, chronic obstructive pulmonary disease (COPD), etc. [4].

CKD is characterized by impairment of renal function and its related complications [5]. End-stage renal disease (ESRD), the most severe form of CKD, defined as patients with an estimated glomerular filtration rate (eGFR) < 15 mL/min per 1.73 m^2^, and a need for regular dialysis [5,6]. High prevalence of CKD burdens the financial expenditures and healthcare system in Taiwan and worldwide [7]. CKD is associated with persistent and chronic systemic inflammation which increases the risks of CVD, especially in patients with diabetes [8]. Meanwhile, CVD is the most common cause of mortality in patients with CKD.

The association between periodontitis and CKD has been reported over the last decade [9,10]. Both diseases have chronic inflammatory characteristics and share some common risk factors [10]. Elevated systemic inflammatory cytokines, such as Interleukin-1β (IL-1β), Interleukin-6 (IL-6), C-Reactive protein (CRP), and Tumor necrosis factor-α (TNF-α), were attributable to periodontitis have been documented by previous studies [11,12]. It could cause vascular endothelial dysfunction and further cardiovascular events [13,14]. A recent consensus report suggests that periodontal treatment could decrease systemic inflammatory response and incidence of cardiovascular events [13].

Until now, limited randomized-controlled clinical trial (RCT) studies comparing the outcomes in patients with ESRD after either non-surgical periodontal treatment or none have been published [15,16]. Much uncertainty still exists in the relationship between the intervention of periodontitis and risk of CKD complications. Therefore, in this study, we aimed to investigate the clinical, biochemical, and microbiological effect of non-surgical periodontal treatment in patients with periodontitis and ESRD.

## 2. Materials and Methods

### 2.1. Study Design

This is a 3-month pilot study conducted in Taipei Medical University Hospital (TMUH) and TMU-affiliated Shuang-Ho Hospital (TMU-SHH) from September 2017 till December 2019. For allocation of included participants, randomization was achieved by a computer-generated list. Only clinicians and study staff members were aware of group assignment, i.e., treatment group or control group. Ethical approval was obtained from TMU-JIRB, Taipei Medical University (N201604007). The study was conducted in accordance with the Helsinki Declaration, following CONSORT guidelines.

### 2.2. Periodontal Participants

As a preliminary study, all hemodialysis patients who were well-informed and signed their informed consent at the hemodialysis department of two hospitals were sent to dental screening for eligibility. Inclusion criteria were as follows: maintained hemodialysis thrice a week for at least 3 months [17]; aged 20 or more than 20; at least 5 natural retainable teeth; and generalized moderate to severe periodontitis according to AAP/CDC criteria [18]. Exclusion criteria were: active infection under antibiotic treatment within the past 4 weeks for preventing the influence of antibiotics to the baseline condition [19]; unable to receive dental treatment or complete the entire course of the study. Patients with other systemic diseases, such as diabetes or cardiovascular disease, were not excluded from this study. We collected the demographics and medical information from the electronic records in the two hospitals. The medical information included baseline blood pressure, body mass index (BMI), dialysis vintage, past medical history, smoking, and laboratory results.

### 2.3. Periodontal Examination

The participants underwent a full periodontal examination at the baseline and after 3 months. An additional periodontal examination for treatment group was performed 4 to 6 weeks after treatment. The following 5 periodontal parameters were collected: plaque index, periodontal probing depth (PD), gingival recession depth (REC), clinical attachment loss (CAL), bleeding on probing (BOP). Six measurement sites per tooth for plaque index, PD, REC, CAL, and BOP were recorded. The periodontal probe (PQ-W, Hu-Friedy, Chicago, IL, USA) was used for periodontal examination by two experienced periodontists.

### 2.4. Periodontal Intervention

Subjects in both groups received data collection and oral hygiene instruction at the baseline and at 3 months. Amoxicillin (2 g) for infection prophylaxis was prescribed prior to periodontal examination and treatment appointments [20]. In the treatment group, subjects received standard non-surgical periodontal therapy for removing supragingival and subgingival biofilm and dental calculus by certified periodontists. Re-evaluation of periodontal condition was carried out 4 to 6 weeks after treatment. In an attempt to eradicate or reduce periopathogenic bacterial levels in remaining deep pockets, subgingival minocycline gel (Sun Star, Osaka, Japan) delivery was conducted in sites of periodontal pocket depth remaining ≥ 5 mm [21].

### 2.5. Biochemical Examination

Blood samples were drawn for blood chemistry analysis. Inflammatory biomarkers including high-sensitivity CRP (hsCRP), TNF-α, IL-1β, IL-6, Pentraxin 3 (PTX3) in serum and gingival crevicular fluid (GCF) were measured at baseline and 3-month visits [22,23]. GCF samples were collected by using Periopaper strips (HARCO Electronics, Irvine, CA, USA) from the deepest periodontal pocket. According to the method of our previous study [24], prior to sampling, the test area was air-dried and isolated with gauze. The first paper strip was slipped into the gingival pocket 1 mm subgingivally for 5 s and discarded immediately in order to avoid contamination of saliva. A second paper strip was inserted in the selected site for another 30 s. The remaining paper strip was the dipped in a 200-μL phosphate-buffered saline container and stored at −80 °C. Routine blood chemistries were analyzed by a central laboratory in the hospital. The cytokine levels were analyzed by ELISA using commercially available kits (R&D Systems, Minneapolis, MN, USA).

### 2.6. Microbiological Analysis

Subgingival biofilm was sampled from the 3 deepest periodontal sites using sterile periodontal curette at baseline and 3-month visits for both groups. The subgingival biofilm samples were then suspended in TE buffer, vortexed and sonicated. The DNA was extracted from subgingival biofilm samples.

The subgingival biofilm samples were mixed with buffer RDD and Benzonase and incubated at 37 °C for 30 min, followed by digestion with Proteinase K and incubated for 30 min at 56 °C [25,26]. They were then homogenized in Lysis Buffer. DNA purification was performed using a QIAamp DNA Microbiome Kit (Qiagen), per manufacturer instructions. Next, 16S rRNA gene amplification and library construction were performed according to the protocols provided by Illumina. Nextera XT Index kit was used to add the Illumina sequencing adapters and dual–index barcodes to the amplicon targets. Quantification and quality of the sequencing libraries were checked using a QSep100 Analyzer (BiOptic Inc., New Taipei City, Taiwan). Lastly, the libraries were normalized and pooled in an equimolar ratio and sequenced with an Illumina Miseq platform with V3 chemistry.

### 2.7. 16. S rRNA Gene Sequence Analysis

Preprocessing of the raw reads includes the removal of universal primers and low-quality bases using cutadapt (v1.18) [27]. Then, the paired reads were processed and analyzed with the DADA2/phyloseq workflow in the R environment. The taxonomy assignment of the inferred amplicon sequence variants (ASVs) was performed using the SILVA reference database (v132) with a minimum bootstrap confidence of 80 [28]. Multiple sequence alignment of the ASVs was performed with DECIPHER (v2.8.1) and phylogenetic tree was constructed using RAxML (v8.2.11) [29,30]. The frequency table, taxonomy and phylogenetic tree information were used to create a phyloseq object for downstream bacterial community analyses using phyloseq (v1.24.2) [31]. The α-diversity indices were calculated using the estimate richness function from the phyloseq package. The R package GUniFrac was used to calculate the various versions of the UniFrac distances by determining the community dissimilarity between groups (β-diversity) [32]. Microbiota enrichment analysis was conducted using the Linear Dis-criminant Analysis (LDA) Effect Size method and visualized as cladogram by using GraPhlAn [33,34].

### 2.8. Outcome Measurement

Our primary outcomes were all-cause mortality, cardiovascular (CV) events, and infection events. Prespecified secondary outcomes included Hemoglobin A1c (HbA1c), cardiac function, and peripheral vessel evaluation. In addition, periodontal parameters, serum/GCF inflammatory biomarkers, and subgingival microbes were collected.

### 2.9. Statistical Analysis

Baseline clinical characteristics were analyzed by Mann–Whitney U test, except gender and periodontal severity, which were analyzed by Fisher’s exact test. Baseline periodontal parameters, plaque index, PD, REC, CAL, and BOP were analyzed in patient-level by Mann–Whitney U test. Paired *t*-test and Student *t*-test were used for comparing changes in PD, REC, and CAL in site-level between the baseline and 3-month visits, and between treatment and control groups. Primary and secondary outcomes were analyzed by Mann–Whitney U test. For evaluating changes in level of inflammatory biomarkers in serum and GCF, and microbials in subgingival biofilm, Wilcoxon signed-rank test and Mann–Whitney U test were used for intra-group and inter-groups comparison. Weighted UniFrac analyses was used to compare the similarity among microbial community before and after the periodontal treatment in treatment group. All calculations were performed using Graphpad Prism software, version 8.4.0 for Mac (Graphpad software Inc., SanDiego, CA, USA) and Microsoft Office Excel 2016 for Windows (Microsoft Co., Redmond, WA, USA). A *p*-value < 0.05 was considered to indicate statistical significance.

## 3. Results

### 3.1. Study’s Sample and Demographics

**Figure 1:**
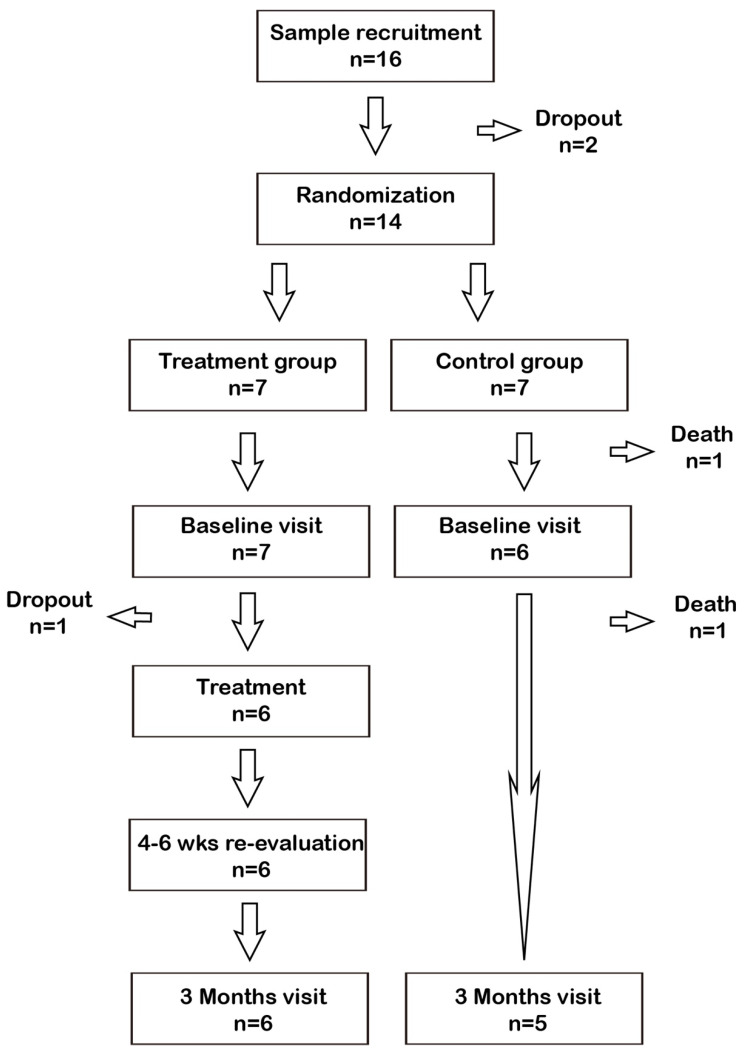
Flow-chart.

A total of 14 eligible ESRD patients consented to participate the study (Figure 1). A total of 11 ESRD patients completed the course of study (Treatment group, *n* = 6; Control group, *n* = 5). After randomization, there were two patients who died in the control group before any intervention and comparison. Clinical characteristics are summarized in Table 1. The mean age was 61 ± 12 years and 71% of the patients were men. 64% of the patients had a history of diabetes and 86% of the patients had severe periodontitis. The clinical parameters were similar between groups, including BMI, systolic blood pressure, periodontitis severity, dialysis vintage, and laboratory results. Baseline periodontal characteristics are summarized in Table 1.

### 3.2. Primary Outcome and Secondary Outcomes

**Figure 2:**
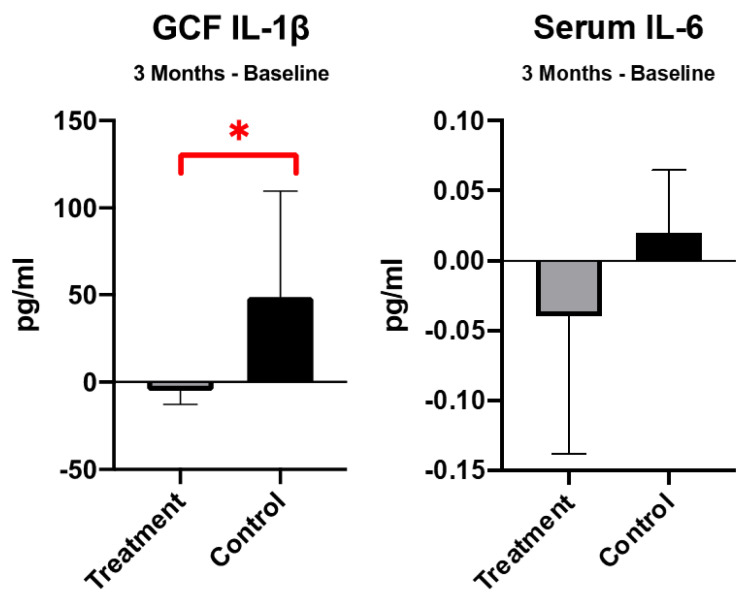
Change of biomarkers level in gingival crevicular fluid (GCF) and serum. *: *p*-value < 0.05.

At 3 months, no significant difference was found between groups in all-cause mortality (OR 0.15, 95% CI 0.01–3.71, *p* = 0.244), CV events (OR 0.29, 95% CI 0.01–8.39, *p* = 0.470), and infection events (OR 1.88, 95% CI 0.20–17.27, *p* = 0.579) (Table 2). In the secondary outcomes, there was no significant difference in either diabetic control, cardiac function, or peripheral vessel evaluation. In the biomarkers, the IL-1β in GCF was significantly reduced in the treatment group as compared to control group. Meanwhile, the IL-6 in serum was non-significantly reduced in the treatment group (Figure 2). Otherwise, other cytokines, including hsCRP, TNF-α, and PTX3 were not significantly changed between groups.

### 3.3. Periodontal Outcomes

**Figure 3:**
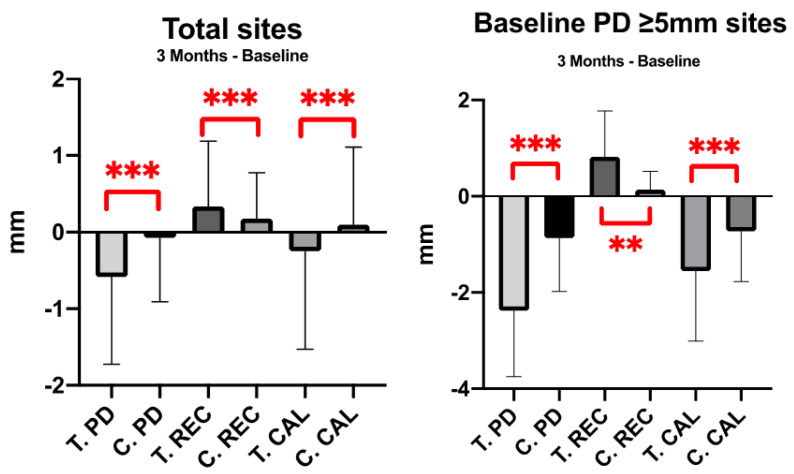
Change of periodontal parameters by site-level analysis. **: *p*-value < 0.003; ***: *p*-value < 0.001.

At patient-level analysis, no significant difference was shown in changes in plaque index, PD, REC, CAL, BOP in intra-group and inter-group comparisons. The exception was the change in REC in treatment group, the gingival recession increased 0.03 mm after treatment (*p* < 0.05). At site-level analysis, changes in PD, REC, and CAL showed significant difference in both groups at the 3 month visit (*p* < 0.001) (Figure 3). There was net attachment gain of 0.25 mm attachment in treatment group, and net attachment loss of 0.1 mm in control group. The significant improvement in PD, REC, and CAL changes were shown in treatment group. Specifically, in severe periodontitis sites (PD ≥ 5 mm sites), the improvement was more obvious in treatment group than control group (Figure 3).

### 3.4. Periodontal Microbiome

**Figure 4:**
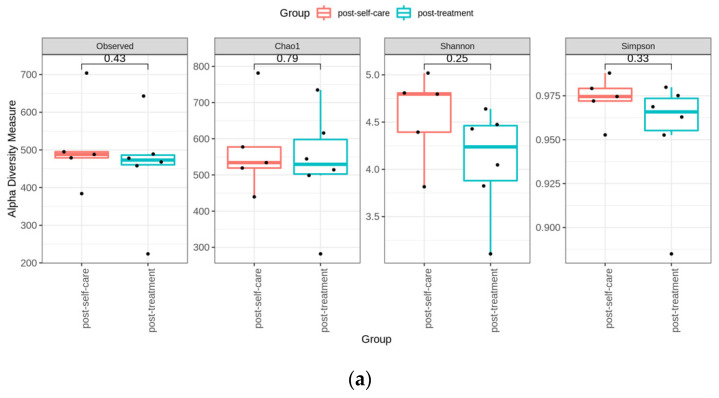

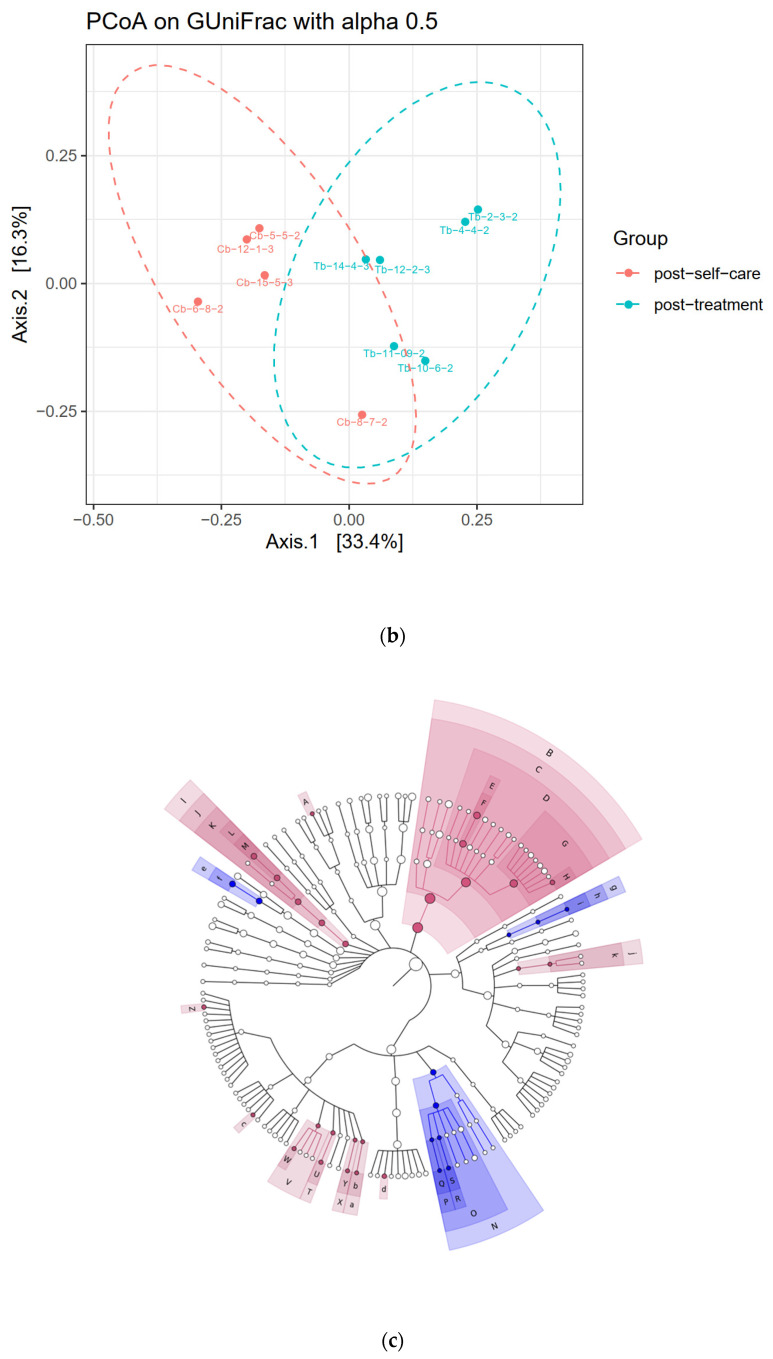

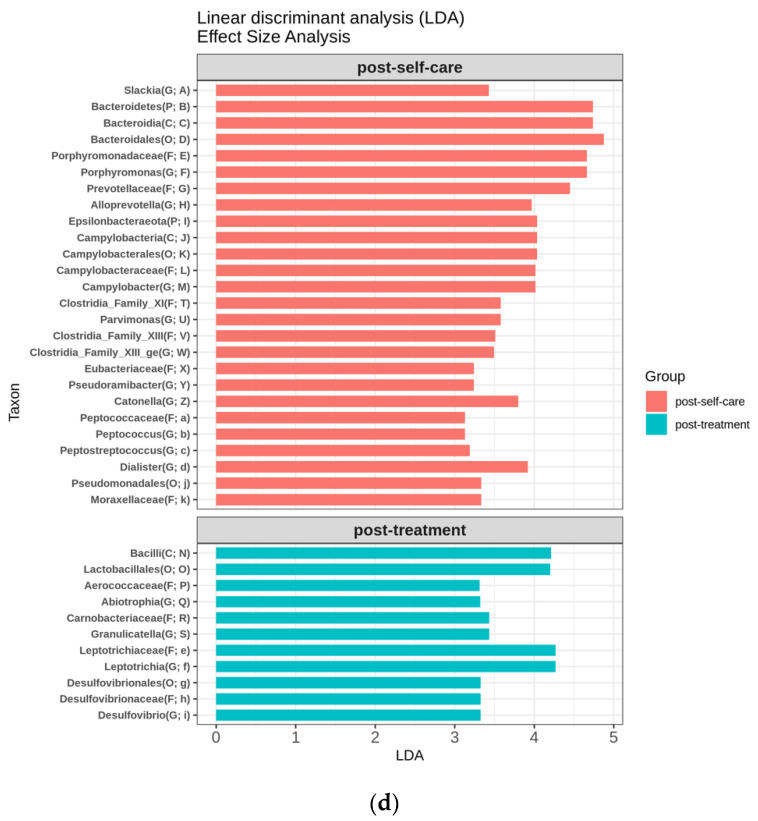
Subgingival microbial diversity. (**a**) Comparison of the α-diversity between the microbiota of the treatment and control group patients. We used the four indices to represent the α-diversity (the observed, Chao, Shannon, Simpson index). (**b**) Principal coordinate analysis. It showed the grouping patterns of the microbiota between the post-treatment and post self-care patients based on the GUniFrac distances, which showed a significant difference between groups (ADONIS analysis, *p* = 0.006). (Each closed circle represents a sample. Distances between any pair of samples represent their dissimilarities.) (**c**) Significantly discriminative taxa between groups were determined using the Linear Discriminant Analysis (LDA) Effect Size. (**d**) LDA shows the taxa meeting the thresholds (>3). (Different colored regions represent different groups. From the interior to the exterior, each layer represents the phylum, class, order, family, and genus level).

We applied α-diversity indices to determine the ecological diversity of the microbial community. Comparison of α-diversity between the two groups was performed. Four indices were not significantly different between groups (Figure 4a). Further, we used the principal coordinate analysis (PCoA), which is a dimensional reduction method to illustrate the relationship between samples, depending on the distance matrix. GUniFrac distance analysis was performed and showed a significant difference between groups (ADONIS analysis, *p* = 0.006) (Figure 4b). Significantly discriminative taxa between groups were determined using the LDA Effect Size (Figure 4c). LDA shows only the taxa meeting the thresholds (>3) (Figure 4d).

The red complex periodontal pathogens, *T. forsythia*, and *T. denticola*, showed significant decrease after periodontal treatment (*p* < 0.05). Bacterial count of *P. gingivalis* and *A. actinomycetemcomitans* were decreased in both groups, but intra-group comparisons did not show significant difference. *A. naeslundii* amount was elevated in treatment group and decreased in control group; however, the difference did not show statistical significance. Inter-group comparison did not show significant difference.

## 4. Discussion

Periodontal disease is a kind of chronic inflammation disease and highly prevalent in dialysis patients [35]. A previous prospective observational study indicated that compared to patients with mild or no periodontitis, all-cause mortality is higher in patients with moderate and severe periodontitis [36]. Another retrospective study with 168 patients under hemodialysis showed moderate to severe periodontitis was associated with increased risk of CV mortality, as compared to those with mild periodontitis [37]. However, the evidence of periodontal treatment reducing mortality in dialysis patients is still insufficient. Based on the present pilot study, periodontal non-surgical treatment group did not show significant difference in primary outcomes including all-cause mortality, CV events, and infection events.

Increased IL-1β in GCF has been considered as a sign of development of periodontal disease. Periodontitis might aggravate systemic inflammatory response [38]. A recent systematic review concluded that non-surgical periodontal treatment can reduce serum hsCRP levels in hemodialysis and/or peritoneal dialysis patients, but did not change IL-6 or albumin levels significantly [15]. However, in our study, even IL-1β in local GCF was significantly reduced in the treatment group, changes in serum level of IL-6, hsCRP, TNF-α, and PTX3 did not show significant difference between treatment and control groups.

Several pre-specified outcome measurements, including sugar change and vascular function, were performed in the present study. A previous review reported chronic periodontitis results in increased expression of pro-inflammatory cytokines, and vascular endothelial dysfunction [39]. A meta-analysis reported the association of periodontitis with carotid intima-media thickness and flow-mediated dilation [40]. The periodontal treatment improved the flow-mediated dilation, which indicated an improvement in endothelial function [40]. However, in our study, all of these outcomes showed no difference between groups during follow up. This result may be explained by the fact that most of the collected studies in the previous meta-analysis study were from patients with normal renal function [40]. Several lines of uremic toxins and uremic milieu effects on the vascular endothelial cell may not be improved dramatically with the locally periodontal inflammation improvement [41].

More than 1000 bacterial species host in the oral cavity [42]. Red complex bacterial species, constituted by *P. gingivalis*, *T. denticola* and *T. forsythia*, are considered as the most virulent periodontal pathogens [43]. In a previous study comprising of 32 periodontitis subjects treated by non-surgical periodontal therapy and maintained for 12-month period, *A. naeslundii* was increased significantly, and the red complex periodontal pathogens were decreased significantly in prevalence and levels after therapy [44]. Our findings are consistent with previous studies, periodontal pathogens were also reduced in treatment group after therapy. Microbial diversity showed significant difference between post-treatment and post self-care groups.

In the present study, all of the periodontal exams were performed by two experienced dentists. Microbiological changes in GCF sampling can also confirm the clinical effects of periodontal treatment, reducing the risk of clinical inconsistency. However, our study has several limitations. First, limited subjects enrolled in the study could diminish the outcomes. Second, differences in mortality would be apparent over a longer duration of time. Third, some possible confounding factors, such as diabetes and smoking, might have influenced the outcomes. Further research should be undertaken to investigate the systemic effects of periodontal treatment on more CKD patient numbers and for longer follow-up period. In clinical practice, clinicians could take periodontal health into consideration in dialysis patients.

## 5. Conclusions

In conclusion, the periodontal non-surgical intervention changes the pattern and distribution of microbiota in gingival sulcus. However, no significant association of the intervention in periodontitis and mortality improvement in dialysis patients was found in present study. Moreover, treatment of periodontitis did not bring beneficial effect in reducing systemic inflammation.

## Figures and Tables

**Table 1:** Baseline clinical and dental characteristics.

	All (*n* = 14)	Treatment (*n* = 7)	Control (*n* = 7)	*p* Value
Male, *n* (%)	10 (71%)	5 (71%)	5 (71%)	0.721
Age	61 ± 12	63 ± 8	60 ± 16	0.675
BMI	22.8 ± 3.7	22.5 ± 2.0	23.2 ± 5.0	0.751
SBP (mmHg)	145 ± 17	137 ± 21	152 ± 9	0.093
DBP (mmHg)	76 ± 16	67 ± 9	86 ± 15	0.014
Severity				1.000
Mild	0	0	0	
Moderate	2	1	1	
Severe	12	6	6	
Dialysis vintage (months)	46 ± 59	60 ±81	31 ± 20	0.381
Diabetes, *n* (%)	9 (64%)	5 (71%)	4 (57%)	0.577
Hypertension, *n* (%)	12 (86%)	5 (71%)	7 (100%)	0.127
Smoking, *n* (%)	0 (0%)	1 (14%)	2 (29%)	0.213
Alb (g/dL)	4.3 ± 0.4	4.3 ± 0.4	4.2 ± 0.4	0.597
Hb (g/dL)	11.4 ± 0.9	11.8 ± 0.8	11.0 ± 0.8	0.084
K (mg/dL)	4.7 ± 0.5	4.8 ± 0.5	4.5 ± 0.4	0.395
Ca (mg/dL)	9.2 ± 0.9	9.1 ± 0.8	9.4 ± 1.1	0.534
P (mg/dL)	5.2 ± 1.2	5.1 ± 1.0	5.2 ± 1.4	0.914
PTH (pg/mL)	310 ± 259	252 ± 175	368 ± 328	0.423
Fe (ug/dL)	84 ± 37	82 ± 23	85 ± 48	0.870
TIBC (ug/dL)	227 ± 33	233 ± 38	221 ± 28	0.501
Ferritin (ng/mL)	570 ± 367	608 ± 439	540 ± 349	0.804
HbA1c (%)	6.3 ± 1.4	6.8 ± 1.7	5.7 ± 0.7	0.301
**Periodontal characteristics (patient-level analysis)**				
Plaque index (%) (mean ± SD) (%)		99.57 ± 0.79	88.14 ± 20.48	0.08
Probing depth (PD) (mm) (mean ± SD)		2.97 ± 0.74	2.99 ± 0.53	0.46
Gingival recession depth (mm) (REC) (mean ± SD)		1.18 ± 0.84	0.83 ± 0.80	0.22
Clinical attachment loss (CAL) (mm) (mean ± SD)		4.18 ± 1.46	3.99 ± 1.31	0.41
Bleeding on probing (BOP) (%) (mean ± SD) (%)		62.29 ± 21.55	77.57 ± 16.33	0.08

Statistics by Mann–Whitney U test for baseline characteristics, except baseline gender and periodontal severity which were by Fisher’s exact test. Statistics by Mann–Whitney U test for baseline plaque index, PD, REC, CAL, BOP. Significance level set at *p*-value < 0.05.

**Table 2:** Primary and secondary outcomes.

	N	Event	OR (95% CI)	*p* Value
Primary outcomes				
All-cause mortality	14	2		0.244
Treatment	7	0	0.15 (0.01–3.71)	
Control	7	2	1.0 (ref.)	
CV events	14	1		0.470
Treatment	7	0	0.29 (0.01–8.39)	
Control	7	1	1.0 (ref.)	
Infection events	14	5		0.579
Treatment	7	3	1.88 (0.20–17.27)	
Control	7	2	1.0 (ref.)	
Secondary outcomes	Treatment (*n* = 6)	Control (*n* = 5)		*p* value
HbA1c	6.6 ± 1.3	5.8 ± 1.2		0.401
Cardiac function				
LVEF	61 ± 17	61 ± 7		0.923
LV mass	281 ± 70	266 ± 58		0.795
Peripheral vessel				
Right ABI	1.1 ± 0.2	1.1 ± 0.1		0.831
Left ABI	1.0 ± 0.2	1.1 ± 0.1		0.097
Right PWV	16.6 ± 3.9	14.6 ± 4.3		0.523
Left PWV	15.4 ± 4.2	15.2 ± 4.7		0.945

Statistics by Mann–Whitney U test. Significance level set at *p*-value < 0.05. LVEF: left ventricular ejection fraction. LV: left ventricular. ABI: ankle-brachial index PWV: pulse wave velocity.

## Data Availability

Data will be available from authors upon reasonable request.
